# Pocket-PROTACs: an interpretable pocket-aware deep learning framework for predicting PROTAC-induced protein degradation

**DOI:** 10.1093/bioinformatics/btag431

**Published:** 2026-08-21

**Authors:** Kai Chen, Zhijian Huang, Yinbo Wang, Siyuan Shen, Jinmiao Song, Lei Deng

**Affiliations:** School of Computer Science and Engineering, Central South University, Changsha, 410083, China; School of Computer Science and Engineering, Central South University, Changsha, 410083, China; School of Computer Science and Engineering, Central South University, Changsha, 410083, China; School of Computer Science and Engineering, Central South University, Changsha, 410083, China; Xinjiang Key Laboratory of Intelligent Computing and Smart Applications, Xinjiang University, Urumqi 830046, China; School of Software, Xinjiang University, Urumqi 830046, China; School of Computer Science and Engineering, Central South University, Changsha, 410083, China; Xinjiang Key Laboratory of Intelligent Computing and Smart Applications, Xinjiang University, Urumqi 830046, China; School of Software, Xinjiang University, Urumqi 830046, China

## Abstract

**Motivation:**

Proteolysis-targeting chimeras (PROTACs) enable targeted protein degradation by recruiting an E3 ubiquitin ligase to a protein of interest (POI) and forming a ternary complex. Despite their therapeutic promise, rational PROTAC design remains challenging, as degradation efficacy depends on subtle and highly structure-dependent interactions among the POI, the E3 ligase, and the bifunctional molecule.

**Results:**

We propose Pocket-PROTACs, a pocket-aware attention-based framework for predicting PROTAC-induced protein degradation from a triplet of POI, E3 ligase, and PROTAC. Pocket-PROTACs encodes protein sequences using a pre-trained protein language model and represents PROTACs with a geometry-aware graph neural network over an ensemble of three-dimensional conformers. Both POI–PROTAC and E3 ligase–PROTAC interactions are explicitly modeled through a residue–atom cross-attention mechanism that captures fine-grained interaction patterns. To improve model interpretability, we introduce a pocket-aware module that incorporates structural context to guide residue-level relevance estimation, enabling multi-level attribution analysis. Experiments on two benchmark datasets show that Pocket-PROTACs consistently outperforms fingerprint-based baselines and recent deep learning methods. The learned relevance maps highlight localized interaction patterns on both the POI and the E3 ligase that are qualitatively consistent with known pocket-level features. A case study on kelch domain containing 2 (KLHDC2)-engaging bromodomain and extra-terminal domain (BET) PROTACs further demonstrates that our model accurately predicts degradation behavior and provides biologically meaningful, attention-based interpretations, offering practical support for PROTAC design and experimental investigation.

**Availability and implementation:**

Source code and datasets are available at https://github.com/Adochew/Pocket-PROTACs.

## 1 Introduction

Proteolysis-targeting chimeras (PROTACs) have emerged as a transformative modality in targeted protein degradation (TPD), providing a powerful strategy to eliminate disease-relevant proteins that are inaccessible to conventional small-molecule inhibition (Békés *et al.* 2022). Unlike traditional inhibitors, which rely on sustained occupancy of a ligandable binding site and are often ineffective against proteins lacking well-defined pockets or harboring resistance mutations, PROTACs function through an event-driven mechanism (Neklesa *et al.* 2017). A PROTAC is a heterobifunctional molecule composed of a ligand targeting the protein of interest (POI) and a ligand recruiting an E3 ubiquitin ligase, connected by a flexible linker (Troup *et al.* 2020, Ishida and Ciulli 2021). By simultaneously engaging the POI and an E3 ligase, a PROTAC promotes formation of a ternary complex, enabling ubiquitination of the target protein and its subsequent proteasomal degradation (Lin *et al.* 2025). This catalytic process allows a single PROTAC molecule to trigger the degradation of multiple POI molecules in a sub-stoichiometric manner (Neklesa *et al.* 2017, Ward *et al.* 2023). As a result, PROTACs can overcome limitations of classical inhibitors, including poor druggability and acquired resistance, thereby expanding the therapeutic landscape (Gadd *et al.* 2017, Békés *et al.* 2022). These advantages have fueled rapid progress in the field, with more than 30 PROTAC candidates entering human clinical trials by 2025, providing compelling evidence of clinical potential (Berkley *et al.* 2025).

Rational PROTAC design remains highly challenging due to the multifaceted and cooperative nature of PROTAC-mediated degradation (Gharbi and Mercado 2024). Successful degraders require coordinated optimization of target and E3 ligase selection, productive ternary complex formation, linker composition, and favorable cellular permeability and stability (Gadd *et al.* 2017). In practice, many PROTACs with high binary binding affinity fail to induce degradation due to suboptimal ternary complex geometry or insufficient cellular exposure (Němec *et al.* 2022), motivating the development of computational methods to predict PROTAC-induced degradation in silico (Park and Jeon 2025).

Existing computational models, however, still face notable limitations in their treatment of structural information. Early methods largely extended classical drug–target interaction frameworks to the PROTAC setting while neglecting three-dimensional structure and binding-site context (Li *et al.* 2020). More recent studies have incorporated structural features—such as representing ligand-binding pockets as graphs (Li *et al.* 2022) or predicting ternary complex stability via deep learning (Heinrich *et al.* 2018)—but typically encode proteins and PROTACs independently, restricting fine-grained cross-modal interaction learning. Structure-based pipelines that explicitly model ternary complex geometries (Marzella *et al.* 2022, Guedeney *et al.* 2023) offer geometric detail but are constrained by the scarcity of high-quality ternary structures and uncertainty from docking assumptions. Interpretability further complicates PROTAC activity prediction, as many deep learning models function as black boxes. Recent attention-based models enable atom- or residue-level attribution (Cai *et al.* 2024, Chen *et al.* 2025), yet still rely on whole-sequence or coarse structural representations rather than explicitly modeling ligand-binding pockets and interaction interfaces. As a result, existing methods lack pocket-aware modeling, effective cross-modal interaction learning, and mechanistically grounded interpretability.

To address these challenges, we propose *Pocket-PROTACs*, an interpretable pocket-aware framework for predicting PROTAC-induced protein degradation. Unlike prior approaches that treat proteins as global entities, Pocket-PROTACs explicitly incorporates ligand-binding pocket residues from the target protein and interface-related residues of the recruited E3 ligase, situating prediction within the spatial context of ternary complex formation and ubiquitination (Sakamoto *et al.* 2003). A residue–atom cross-attention mechanism further enables fine-grained cross-modal interaction learning between pocket-level protein residues and atom-level PROTAC regions, yielding residue-level importance scores that provide mechanistically interpretable insights into degradation prediction.

Overall, Pocket-PROTACs bridges predictive performance and interpretability in PROTAC-induced protein degradation. Extensive experiments demonstrate that our framework achieves robust and competitive performance on two benchmark datasets. Importantly, the pocket-aware attention mechanism enables residue-level analysis of PROTAC interactions with both the POI and the E3 ligase, facilitating the identification of structurally and biologically relevant regions that may govern degradation. By integrating pocket-aware modeling, cross-modal interaction learning, and interpretable evidence extraction, Pocket-PROTACs offers a practical and transparent framework for structure-guided analysis and rational PROTAC design.

## 2 Materials and methods

### 2.1 Overview

**Figure 1:**
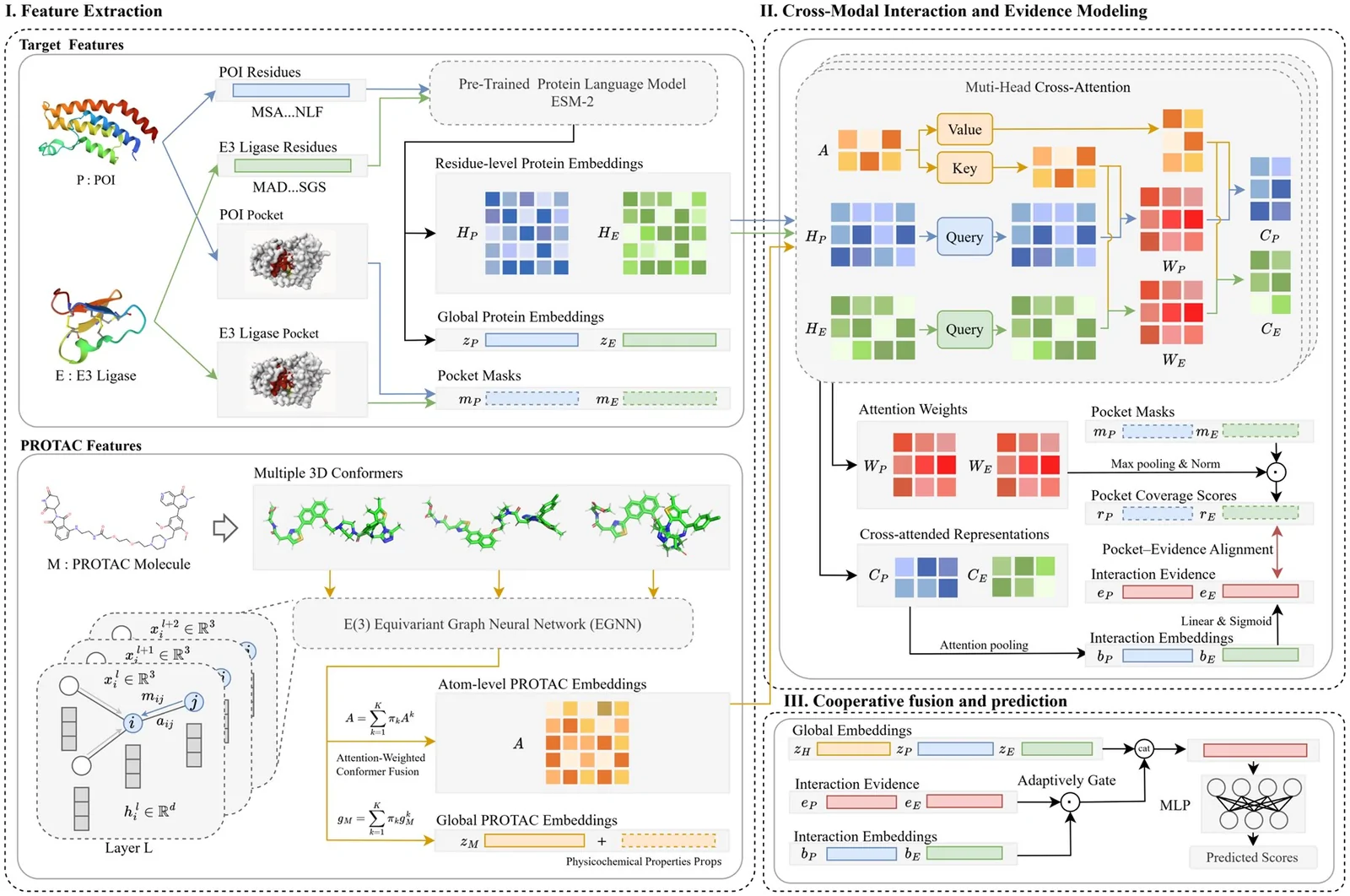
Overview of the Pocket-PROTACs framework. (I) Feature extraction: POI and E3 ligase sequences are encoded by a protein language model with pocket annotations, while PROTAC molecules are represented by 3D conformer ensembles. (II) Cross-modal interaction modeling: POI–PROTAC and E3–PROTAC interactions are captured via multi-head cross-attention to derive pocket-level interaction embeddings and evidence. (III) Fusion and prediction: interaction embeddings and evidence are fused with global representations to predict degradation.

The overall architecture of Pocket-PROTACs is illustrated in Fig. 1. Given an input triplet (P,E,M), the model first encodes the POI and the E3 ligase using a pre-trained protein language model to obtain residue-level embeddings and global protein representations. In parallel, the PROTAC molecule is represented by a geometry-aware graph neural network applied to an ensemble of three-dimensional conformers, producing atom-level features and a global molecular embedding.

Protein and molecular representations are then coupled through cross-modal attention to model interactions between protein residues and molecular substructures. This stage yields compact interaction embeddings for both proteins together with residue-level importance scores. These scores are further summarized with respect to predefined or predicted pocket regions to obtain pocket-consistent interaction evidence. Finally, the interaction evidence is fused with the global representations of the POI, E3 ligase, and PROTAC molecule, and the resulting representation is passed to a prediction network to produce the degradation probability $\hat{y}$.

### 2.2 Protein representation via pre-trained models

To obtain informative protein representations without relying on explicit 3D structures, Pocket-PROTACs employs a pre-trained protein language model (PLM) to encode both the POI *P* and the E3 ligase *E*. Such models leverage large-scale sequence data to learn embeddings that implicitly capture structural and functional regularities, making them particularly suitable for PROTAC settings where high-quality ternary complex structures are scarce.

Given the amino-acid sequence of *P*, the PLM produces a residue-level embedding matrix $H_{P}\in R^{L_{P}\times 1280}$ and a global sequence embedding $z_{P}\in R^{1280}$, where $L_{P}$ is the sequence length. Analogous representations $H_{E}$ and $z_{E}$ are obtained for *E*. These high-dimensional embeddings are projected to the model hidden dimension *d* via linear transformations:


(1) $$H_{X}^{d}=\text{LN}(H_{X}W_{\text{res}}),\;z_{X}^{d}=z_{X}W_{z},\;X\in \{P,E\},$$

where $W_{\text{res}},W_{z}\in R^{1280\times d}$ are learnable projection matrices and LN(·) denotes layer normalization.

### 2.3 PROTAC molecular representation via conformer ensemble EGNN

PROTAC molecules are highly flexible and may adopt multiple conformations when forming productive ternary complexes. To account for this flexibility, Pocket-PROTACs represents each molecule *M* as an ensemble of *K* low-energy conformers generated by standard conformer sampling procedures. Each conformer is modeled as an atom-level graph with node features derived from atomic identities and 3D coordinates.

An equivariant graph neural network (EGNN) is applied to each conformer to jointly update atomic features and coordinates in a geometry-aware manner, yielding atom-level embeddings that capture both chemical and spatial information (Liu *et al.* 2023). Each conformer is summarized into a global embedding via mean pooling, and an attention mechanism is introduced to weight conformers according to their relative importance:


(2) $$\pi_{k}=\frac{\;\text{exp}(u^{⊤}g_{M}^{\left(k\right)}/T)}{\sum_{j=1}^{K}\;\text{exp}\;(u^{⊤}g_{M}^{\left(j\right)}/T)},$$

where $g_{M}^{\left(k\right)}$ denotes the global embedding of the *k*-th conformer, u is a learnable vector, and *T* is a temperature parameter. The final molecular representation is obtained by aggregating atom-level and global conformer embeddings using attention weights.

In addition to structural information, physicochemical descriptors are incorporated to complement the geometric features. The aggregated molecular embedding and the projected property features are concatenated and transformed to form the final global PROTAC representation $z_{M}$.

### 2.4 Cross-attention for interaction modeling

Given the projected residue-level protein representations $\left\{H_{P}^{d},H_{E}^{d}\right\}$ and the atom-level PROTAC representation *A*, Pocket-PROTACs models protein–molecule interactions via cross-attention (Hou *et al.* 2019), allowing residues to selectively attend to molecular substructures and capture residue–ligand correspondence patterns relevant to ternary complex formation.

Two parallel cross-attention modules are employed for POI–PROTAC and E3–PROTAC interactions, where residues act as queries and PROTAC atoms serve as keys and values:


(3) $$C_{P}=\text{Attn}\left(H_{P}^{d},A,A\right),\;C_{E}=\text{Attn}\left(H_{E}^{d},A,A\right),$$

where Attn(·) denotes a multi-head scaled dot-product attention operation, and $C_{P}\in R^{L_{P}\times d}$ and $C_{E}\in R^{L_{E}\times d}$ are cross-attended residue representations conditioned on the PROTAC structure.

To obtain compact interaction representations for downstream prediction, residue-level features are aggregated using a learnable attention pooling mechanism. Specifically, pooling weights $\alpha_{P,i}$ and $\alpha_{E,i}$ are obtained by applying a scoring function followed by softmax normalization over residues:


(4) $$b_{P}=\sum_{i=1}^{L_{P}}\alpha_{P,i}C_{P,i},\;b_{E}=\sum_{i=1}^{L_{E}}\alpha_{E,i}C_{E,i},$$

where $\alpha_{P,i}$ and $\alpha_{E,i}$ are normalized pooling weights computed over residues. In addition, cross-attention weights provide residue-level importance scores $a_{P}\in R^{L_{P}}$ and $a_{E}\in R^{L_{E}}$, which indicate the relative contribution of individual residues to POI–PROTAC and E3–PROTAC interactions. Further implementation details of the cross-attention mechanism and residue importance modeling are provided in the Supplementary Information, available as supplementary data at *Bioinformatics* online.

### 2.5 Evidence modeling and cooperative fusion

Although cross-attention captures detailed residue–molecule interaction patterns, not all learned interactions are equally reliable or relevant to productive ternary complex formation. To explicitly model interaction reliability and support mechanistically grounded interpretation, Pocket-PROTACs introduces an evidence modeling mechanism. Given the pooled interaction embeddings $b_{P},b_{E}\in R^{d}$, we derive confidence-aware evidence vectors $e_{P},e_{E}\in {\left(0,1\right)}^{d}$ using a learnable transformation followed by element-wise sigmoid gating. Each dimension of $e_{P}$ and $e_{E}$ represents soft evidence supporting the corresponding POI–PROTAC or E3 ligase–PROTAC interaction feature.

To align learned interaction evidence with biologically plausible binding regions, we incorporate a pocket-aware alignment mechanism. Residue-level importance vectors $a_{P}\in R^{L_{P}}$ and $a_{E}\in R^{L_{E}}$, derived from cross-attention weights, quantify how strongly individual residues participate in POI–PROTAC and E3 ligase–PROTAC interactions. Using predefined or predicted pocket masks $m_{P}$ and $m_{E}$, we summarize the concentration of attention within the pocket as


(5) $$r_{P}=\sum_{i=1}^{L_{P}}a_{P,i}m_{P,i},\;r_{E}=\sum_{i=1}^{L_{E}}a_{E,i}m_{E,i}.$$

During training, we enforce consistency between evidence strength and pocket-localized attention via a pocket alignment loss:


(6) $$L_{\text{align}}={\left(\bar{e_{P}}-r_{P}\right)}^{2}+{\left(\bar{e_{E}}-r_{E}\right)}^{2},$$

where $\bar{e_{P}}$ and $\bar{e_{E}}$ denote the mean evidence values.

Beyond interpretability, evidence modeling also modulates cooperative interaction strength between the POI and E3 ligase. Specifically, the interaction embeddings $b_{P}$ and $b_{E}$ are first combined, and their contribution is adaptively scaled by the agreement between the evidence vectors $e_{P}$ and $e_{E}$, yielding a gated cooperative interaction embedding *u*. The detailed formulations and implementation details of the evidence modelling and gating strategy are provided in the Supplementary Information, available as supplementary data at *Bioinformatics* online.

Finally, the gated interaction embedding *u* is fused with global protein and molecular context to form the final prediction:


(7) $$t_{0}=\psi \left(\left[z_{P}^{d}\|z_{E}^{d}\|z_{M}\right]\right),\;\hat{y}=\sigma \left(h\left(\left[u\|t_{0}\right]\right)\right),$$

where ψ(·) and h(·) denote multilayer perceptrons. The output $\hat{y}\in (0,1)$ represents the predicted probability that the given PROTAC induces degradation of the target protein.

## 3 Results

### 3.1 Datasets

We constructed two benchmark datasets derived from PROTAC-DB (Weng *et al.* 2021, Ge *et al.* 2025) to evaluate model performance under consistent curation and labeling standards. The first dataset was processed from PROTAC-DB 1.0 (Weng *et al.* 2021). After data cleaning and label assignment, the first dataset contains 1172 unique PROTAC molecules, involving 121 target proteins and 6 E3 ligases, forming 1501 valid PROTAC–target–E3 triplets (577 active, 924 inactive), and is referred to as Protac1501. The second dataset was independently collected and curated by us from the latest version of PROTAC-DB 3.0 (Ge *et al.* 2025). The raw database contains a substantially larger but more heterogeneous set of entries with frequent missing annotations. After applying the same cleaning and labeling pipeline, the second dataset consists of 2837 unique PROTAC molecules, 199 target proteins, and 10 E3 ligases, corresponding to 3656 well-defined triplets (2381 active, 1275 inactive), and is referred to as Protac3656. For each retained triplet, binding-pocket information was incorporated to support pocket-aware modeling. When experimentally annotated pocket residues were available from PDB structures, these were used directly; otherwise, pocket regions were predicted using P2Rank (Krivák and Hoksza 2018).

Data curation ensured the consistency of PROTAC samples, and a molecule-disjoint splitting strategy was adopted to evaluate generalization to unseen compounds, following prior PROTAC degradation prediction studies (Cai *et al.* 2024, Chen *et al.* 2025). All samples associated with the same PROTAC molecule were assigned to either the training or test set (8:2 ratio), and the procedure was repeated with different random seeds. To further assess model generalization under realistic deployment scenarios, we conducted an additional strict cold-start evaluations on the Protac3656 dataset. Under this setting, the PROTAC and targeted protein of the training and test sets are strictly non-overlapping. Compared with the commonly used setting where only PROTAC compounds are required to be non-overlapping between training and test sets, this protocol imposes a stricter constraint. Additional implementation details on data curation, label assignment, and tools are provided in the Supplementary Information, available as supplementary data at *Bioinformatics* online.

### 3.2 Performance evaluations

Table 1 summarizes the predictive performance on Protac1501 and Protac3656 under the same 8:2 split, where test PROTAC compounds are completely excluded from training. The baselines include classical machine learning methods, a SMILES-based Transformer, and recent PROTAC-specific deep learning models (ET-PROTACs and PROTAC-STAN). To ensure a fair comparison, all baseline methods, including ET-PROTACs and PROTAC-STAN, were trained from scratch on our curated datasets using identical data splits, evaluation protocols, and random seeds. All results reported in Table 1 are averaged over five independent random splits. Overall, Pocket-PROTACs consistently achieves the best or near-best performance across all evaluation metrics, demonstrating clear advantages over both fingerprint-based models and existing PROTAC-oriented frameworks.

**Table 1:** Performance comparison of different methods on the Protac1501 and Protac3656 datasets.^a^

Dataset	Method	Fingerprint	AUC	ACC	F1	Pre	Rec
Protac1501	DNN	MACCS	0.7621±0.021	0.7026±0.015	0.6483±0.027	0.6127±0.032	0.6884±0.014
		Morgan	0.7915±0.013	0.7369±0.016	0.6918±0.019	0.6674±0.024	0.7179±0.018
	SVM	MACCS	0.7702±0.019	0.7168±0.016	0.5574±0.018	0.7286±0.027	0.4513±0.022
		Morgan	0.8327±0.011	0.7762±0.014	0.7143±0.012	0.7207±0.025	0.7080±0.018
	RF	MACCS	0.8532±0.013	0.7762±0.010	0.7217±0.022	0.7094±0.017	0.7345±0.015
		Morgan	0.8604±0.012	0.7727±0.013	0.6977±0.017	0.7353±0.020	0.6637±0.019
	Transformer	MACCS	0.8550±0.028	0.7774±0.016	0.7331±0.020	0.6667±0.020	0.8142±0.028
		Morgan	0.8622±0.023	0.8140±0.016	0.7544±0.017	0.7478±0.019	0.7611±0.027
	ET-PROTACs	–	0.8503±0.023	0.8079±0.019	0.7752 ± 0.020	0.7463 ± 0.028	**0.8511** ± **0.026**
	PROTAC-STAN	–	0.8732 ± 0.052	0.8164 ± 0.050	0.7726±0.071	0.7403±0.025	0.8092±0.028
	Pocket-PROTACs	–	**0.8902** ± **0.021**	**0.8272** ± **0.017**	**0.7874** ± **0.024**	**0.7502** ± **0.055**	0.8359 ± 0.053
Protac3656	DNN	MACCS	0.7812±0.013	0.7486±0.014	0.8124±0.015	0.7841±0.021	0.8429±0.016
		Morgan	0.8237±0.012	0.7819±0.012	0.8368±0.006	0.8113±0.013	0.8640±0.011
	SVM	MACCS	0.7396±0.007	0.7199±0.008	0.8079±0.019	0.7483±0.008	0.8778±0.018
		Morgan	0.8326±0.005	0.7883±0.007	0.8467±0.011	0.8231±0.016	0.8717±0.016
	RF	MACCS	0.8582 ± 0.011	0.8019±0.007	0.8456±0.007	0.8465 ± 0.016	0.8447±0.009
		Morgan	0.8354±0.005	0.7964±0.005	0.8490±0.005	0.8448±0.014	0.8534±0.011
	Transformer	MACCS	0.8295±0.014	0.7746±0.009	0.8294±0.014	0.8251±0.020	0.8347±0.013
		Morgan	0.8548±0.013	0.8079 ± 0.008	0.8537 ± 0.011	0.8256±0.013	0.8781 ± 0.007
	ET-PROTACs	–	0.8451±0.015	0.7907±0.010	0.7862±0.015	0.7736±0.020	0.8362±0.019
	PROTAC-STAN	–	0.8058±0.017	0.8046±0.019	0.8496±0.017	0.8360±0.017	0.8581±0.034
	Pocket-PROTACs	–	**0.8844** ± **0.016**	**0.8237** ± **0.011**	**0.8675** ± **0.014**	**0.8500** ± **0.006**	**0.8864** ± **0.034**

^a^ Bold values indicate the best performance and underlined values indicate the second-best performance in each column.

On the smaller Protac1501 dataset, Pocket-PROTACs achieves the highest AUC of 0.8902, outperforming PROTAC-STAN (0.8732, +1.95%) and ET-PROTACs (0.8503, +4.69%). It also yields the best ACC (0.8272) and F1-score (0.7874), improving over PROTAC-STAN by +1.32% and +1.92%, respectively. Although ET-PROTACs attains the highest recall (0.8511), Pocket-PROTACs achieves higher precision (0.7502), resulting in a superior F1-score and AUC. This indicates that Pocket-PROTACs better suppresses false positives under limited data, leading to more balanced and stable predictions in the low-sample regime.

On the larger Protac3656 dataset, Pocket-PROTACs shows more consistent and substantial improvements. It achieves the best AUC of 0.8844, exceeding the strongest fingerprint-based baseline RF+MACCS (0.8582, +3.05%) and ET-PROTACs (0.8451, +4.65%). Pocket-PROTACs also attains the highest ACC (0.8237), improving over PROTAC-STAN (0.8046, +2.37%) and RF+MACCS (0.8019, +2.72%). For F1-score, it reaches 0.8675, outperforming PROTAC-STAN (0.8496, +2.11%) and RF+Morgan (0.8490, +2.18%). Notably, Pocket-PROTACs achieves the highest recall (0.8864) while maintaining strong precision (0.8500), indicating improved sensitivity to degraders without compromising specificity. Compared with Protac1501, the recall gain on Protac3656 suggests that evidence-driven, pocket-aware interaction modeling benefits from increased data diversity, enabling more reliable positive identification. Additional experiments, including the entity-level cold-start evaluation and parameter sensitivity analysis, are provided in the Supplementary Information, available as supplementary data at *Bioinformatics* online.

### 3.3 Interpretability analysis

**Figure 2:**
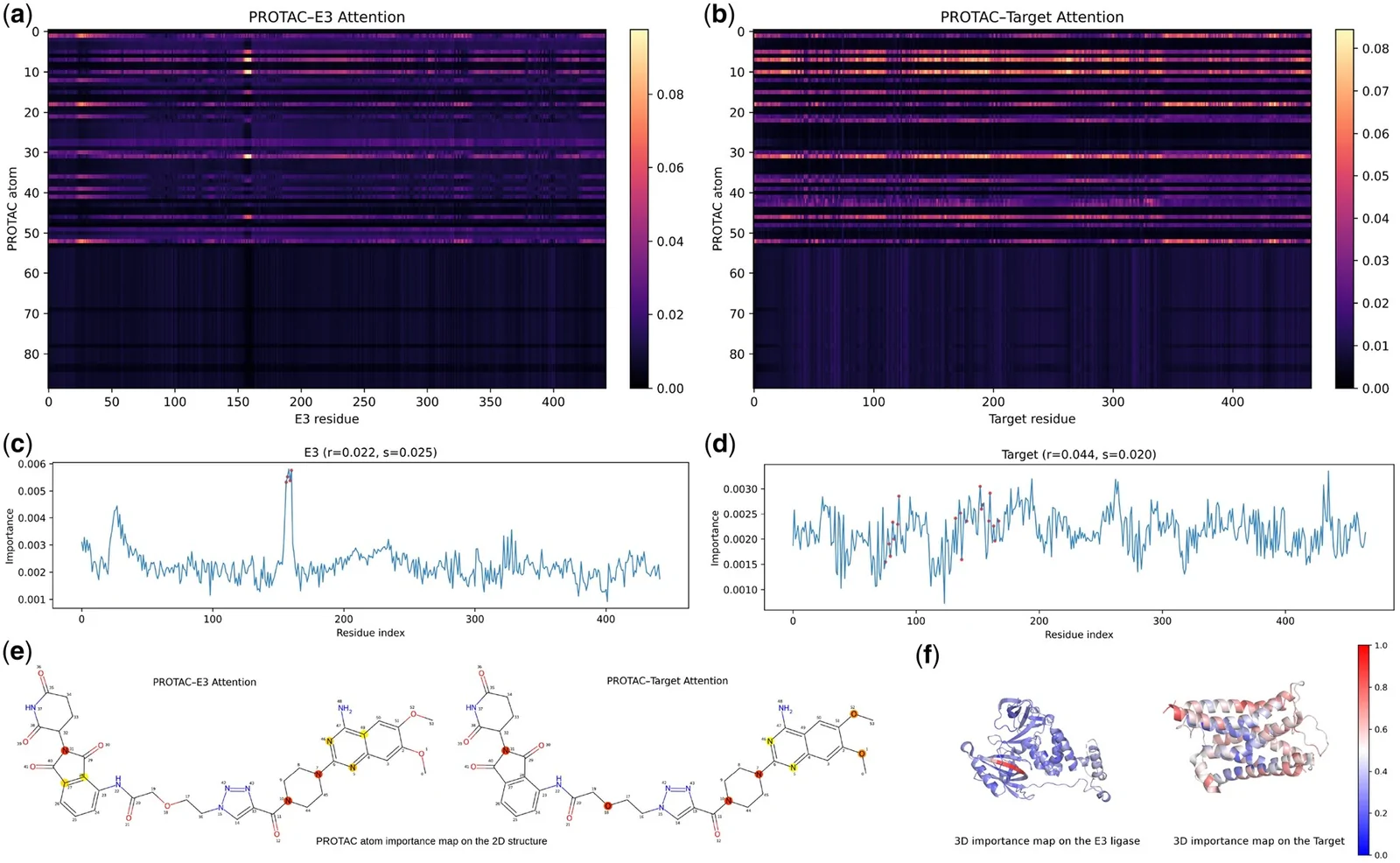
Interpretability analysis of Pocket-PROTACs on a representative test case. (a) Cross-modal attention heatmap between PROTAC atoms and E3 ligase residues. (b) Cross-modal attention heatmap between PROTAC atoms and target (POI) residues. (c) Residue-level importance profile for the E3 ligase obtained by aggregating atom–residue attentions. (d) Residue-level importance profile for the POI obtained similarly. (e) PROTAC atom importance maps shown separately for E3 ligase engagement (left) and POI engagement (right). (f) 3D importance maps projected onto the POI and E3 ligase structures.

To better understand how Pocket-PROTACs makes degradation predictions under the unseen-molecule setting, we conducted an interpretability analysis by examining the learned cross-modal attention and the derived importance signals at the residue and atom levels (Fig. 2). These analyses provide an interpretable view of how different molecular components contribute to PROTAC-induced degradation predictions, offering intuitive insights into target engagement, E3 recruitment, and their coordination within the model.

#### 3.3.1 Cross-attention map analysis of PROTAC–E3 and PROTAC–POI interactions

Figure 2a shows the cross-modal attention map between PROTAC atoms and cereblon (CRBN) residues. The attention distribution is sparse and structured, with clear horizontal bands across specific atom indices and vertical stripes concentrated on a limited number of residues. This pattern indicates that the model repeatedly focuses on a small subset of CRBN residues rather than distributing attention uniformly along the sequence. The aggregated residue-level importance curve in Fig. 2c further highlights this behavior, where a sharp peak emerges at a specific residue region. This observation is consistent with the well-characterized ligand-binding pocket of CRBN in the C-terminal domain, which mediates PROTAC recruitment (Vicente *et al.* 2025). Canonical CRBN recruiters such as IMiDs bind within a conserved hydrophobic pocket through the glutarimide moiety, forming hydrogen-bond and van der Waals interactions with a fixed set of residues. Similar pocket-centered recruitment mechanisms have also been reported for other E3 ligases such as VHL (Hanzl *et al.* 2023). These observations suggest that the sharp signal observed in the CRBN importance profile reflects stable and pocket-centered evidence accumulation on the E3 side.

In contrast, the PROTAC–target attention map in Fig. 2b shows a more distributed pattern. Although attention remains sparse, high-intensity regions extend across multiple residue segments rather than forming a single dominant stripe. This suggests that PROTAC engagement on the POI may involve a broader and more heterogeneous interface compared with E3 recruitment. Such behavior is consistent with recent studies indicating that PROTAC ternary complexes often exhibit conformational flexibility and dynamic reorganization during complex formation (Ma *et al.* 2025). In addition, structural analyses have shown that PROTAC warheads frequently bind shallow grooves or noncanonical surfaces on target proteins rather than deeply buried pockets (Huber *et al.* 2025). Correspondingly, the residue-level importance profile in Fig. 2d displays multiple moderate peaks along the POI sequence, suggesting that the model aggregates interaction evidence from several regions instead of relying on a single residue cluster. This pattern may reflect the diversity of target proteins and the variability of warhead binding interfaces (Troup *et al.* 2020).

#### 3.3.2 Atom-level and residue-level attention analysis

Figure 2e visualizes atom-level importance separately for the E3–PROTAC and POI–PROTAC interaction branches, with the top-12 atoms highlighted per branch. The two maps show distinct but complementary patterns: E3 attention concentrates around the glutarimide-like E3-recruiting moiety, while POI attention is more broadly distributed across the POI-binding warhead and linker-adjacent regions. A partial overlap is observed around linker junction atoms, which occupy a central structural role in the ternary complex and may simultaneously contribute to both interaction branches. Further detailed analysis and additional visualization examples are provided in the Supplementary Information, available as supplementary data at *Bioinformatics* online.

To provide spatial context, residue-level importance scores derived from cross-attention were projected onto the three-dimensional structures of the POI and the E3 ligase (Fig. 2f). The color scale reflects model-assigned relevance, with red, white, and blue indicating high, intermediate, and low importance, respectively. On the POI side, high-importance residues form a contiguous surface region, suggesting that the model focuses on a plausible interaction interface rather than diffuse global signals, consistent with the profile in Fig. 2d. In contrast, the E3 ligase importance map is markedly more localized, with a compact high-importance hotspot aligned with the sharp peak in Fig. 2c, reflecting the structurally conserved nature of E3 ligand-binding pockets. Overall, these visualizations demonstrate that Pocket-PROTACs yields spatially coherent and protein-specific relevance patterns consistent with its attention-based interaction modeling.

## 4 Ablation study

To evaluate the contribution of individual modules in Pocket-PROTACs, we constructed several ablation variants by removing or replacing one component at a time, and report averaged results over repeated runs on the same data split (Table 2). These variants examine the effects of conformer ensembles (*SingleConf*), pocket masks (*NoPocket*), global physicochemical descriptors (*NoProps*), geometry-aware molecular modeling (*GCN-Mol*), cross-modal attention (*BilinearAttn*, *NoAttn*), and protein representations (*kmer-Protein*).

**Table 2:** Ablation study on the Protac3656 dataset.^a^

ID	Variant	AUC	ACC	F1	Pre	Rec
Abl-1	SingleConf	0.9001	0.8106	0.7765	0.7122	0.8534
Abl-2	NoPocket	0.8762	0.8040	0.7631	0.7143	0.8190
Abl-3	NoProps	0.8899	0.8306	0.7884	0.7600	0.8190
Abl-4	GCN-Mol	0.8807	0.7973	0.7749	0.6774	**0.9052**
Abl-5	BilinearAttn	0.8801	0.8073	0.7769	0.7014	0.8707
Abl-6	NoAttn	0.8228	0.7475	0.7099	0.6370	0.8017
Abl-7	kmer-Protein	0.8776	0.8040	0.7531	0.7317	0.7759
Pocket-PROTACs		**0.9090**	**0.8405**	**0.7949**	**0.7686**	0.8230

a Each variant removes or replaces one key component of Pocket-PROTACs. Bold values indicate the best performance and underlined values indicate the second-best performance in each column.

Overall, the results indicate that cross-modal attention and geometry-aware molecular modeling are the most critical factors for performance. Removing attention entirely (*NoAttn*) causes the largest performance degradation, demonstrating the importance of explicitly modeling atom–residue interactions. Simplifying the attention mechanism (*BilinearAttn*) also leads to consistent declines, suggesting that multi-head attention better captures diverse interaction patterns. Replacing EGNN with GCN (*GCN-Mol*) or collapsing the conformer ensemble (*SingleConf*) further reduces performance, highlighting the importance of spatial equivariance and conformational diversity; notably, *GCN-Mol* achieves high recall but lower precision, indicating a tendency to over-predict degraders when spatial constraints are weakened. Removing pocket information (*NoPocket*) and pre-trained protein features (*kmer-Protein*) results in moderate performance drops, confirming their roles as informative structural priors, whereas excluding global physicochemical descriptors (*NoProps*) only slightly affects the results. The gain from conformer ensembles over a single conformer (*SingleConf*) is modest overall; the consistent improvements in ACC, F1, and precision may tentatively suggest better stability for highly flexible PROTAC molecules. These findings collectively demonstrate that the components of Pocket-PROTACs contribute complementary information for accurate degradation prediction. Additional experiments on the contribution of pocket information are provided in the Supplementary Information, available as supplementary data at *Bioinformatics* online.

## 5 Case study

To evaluate model performance on experimentally validated systems, we conducted a case study on kelch domain containing 2 (KLHDC2)-engaging PROTACs reported by Scott *et al.* (2024), which provide a controlled setting for assessing paralog-selective degradation among bromodomain and extra-terminal domain (BET) famly members (BRD2, BRD3, and BRD4). The system includes four structurally related PROTACs (SJ46420, SJ46421, SJ48087, and SJ48088) sharing the same BET-binding warhead and KLHDC2-recruiting ligand but differing in linker composition and terminal modifications. Degradation labels were derived from reported protein abundance measurements, with targets showing less than 50% remaining protein classified as degraded. Additional experimental details and description of the case study are provided in the Supplementary Information, available as supplementary data at *Bioinformatics* online.

**Figure 3:**
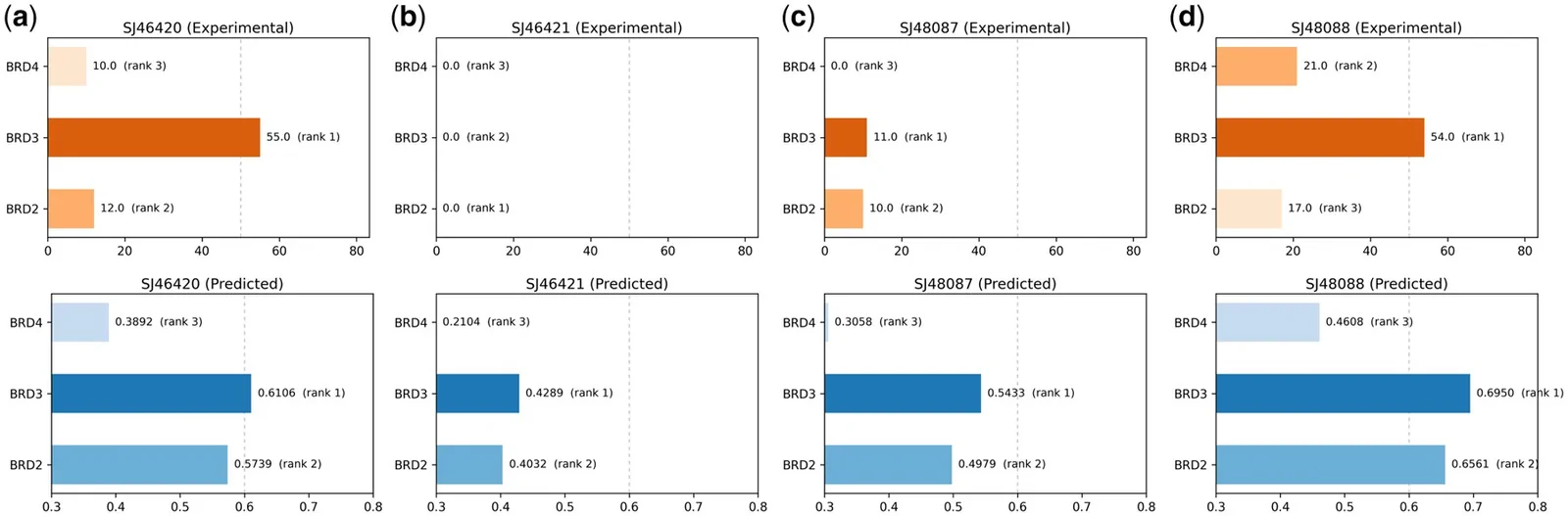
Case study on KLHDC2-based BET PROTACs. For each compound, the upper panel shows experimental degradation extent (%) for BRD2/BRD3/BRD4, and the lower panel shows model-predicted scores. Panels (a): SJ46420; (b): SJ46421; (c): SJ48087; (d): SJ48088. Ranks are annotated for each target.

We applied the proposed model to predict the degradation outcomes of KLHDC2-based PROTAC–target pairs and further evaluated its ability to capture degradation preferences among closely related BET paralogs (BRD2, BRD3, and BRD4), as illustrated in Fig. 3. The dashed vertical lines denote the decision thresholds used for classification, corresponding to 50% degradation extent for experimental measurements and a predicted score of 0.6 for model outputs. Among the 12 evaluated pairs, the model made a single misprediction for the BRD2 target of SJ48088, predicting degradation while experimental measurements indicated a non-degraded state. All other predictions, including those for SJ46420, SJ48087, and SJ46421, were fully consistent with experimentally determined labels.

Beyond classification performance, we further compared experimental degradation extents and model-predicted scores to examine the ranking consistency across BET paralogs. It should be noted that the experimental values represent normalized protein abundance reduction, while model outputs are predicted degradation probabilities that are not directly comparable in absolute terms; the model is designed to capture relative ranking and binary classification outcomes rather than precise degradation magnitudes. For the compounds SJ46420, SJ48087, and SJ48088, the model correctly identifies BRD3 as the most susceptible target. The relative ordering between BRD2 and BRD4 is preserved for SJ46420 and SJ48087. For SJ46421, experimental measurements indicate negligible degradation across all BET paralogs, and correspondingly, the model assigns uniformly low predicted scores, reflecting a consistent assessment of its limited degradation capability. For SJ48088, while BRD3 is correctly ranked first, the ordering between BRD2 and BRD4 is reversed compared with experimental measurements. Overall, these results demonstrate that the proposed model can reliably distinguish degraded from non-degraded targets while capturing paralog-level degradation preferences in this KLHDC2-based system.

## 6 Conclusion

In this work, we introduced Pocket-PROTACs, a pocket-aware and interpretable deep learning framework for predicting PROTAC-induced protein degradation. By explicitly modeling binding-pocket residues and leveraging residue–atom cross-attention, the model captures fine-grained interaction patterns critical for productive ternary complex formation. Experiments on benchmark datasets demonstrate that Pocket-PROTACs consistently outperforms existing machine learning and deep learning baselines while providing residue- and atom-level relevance maps that align with known binding pockets on both the target protein and the recruited E3 ligase. The resulting pocket-aware attention maps highlight key protein residues and PROTAC substructures involved in degradation, offering useful insights for linker optimization, warhead selection, and E3 ligase engagement. Overall, Pocket-PROTACs integrates pocket-level structural awareness, cross-modal interaction modeling, and interpretable attribution, providing a practical computational tool to support structure-guided PROTAC design and optimization as more structural data on protein–PROTAC–E3 complexes become available.

## Supplementary Material

btag431_Supplementary_Data

## Data Availability

The code and data underlying this article are available at https://github.com/Adochew/Pocket-PROTACs.
