# Supplementary material for: Pocket-PROTACs: an interpretable pocket-aware deep learning framework for predicting PROTAC-induced protein degradation
Source: Bioinformatics. 2026 Aug 21;42(Suppl 2):btag431. doi: 10.1093/bioinformatics/btag431 (PMC13501292; doi:10.1093/bioinformatics/btag431)
Supplement: Supplementary file 1 [file btag431_supplementary_data.pdf]

Pocket-PROTACs: deep interpretable learning model with pocket-aware attention for PROTAC-induced protein degradation

Kai Chen [1,†], Zhijian Huang [1,†], Yinbo Wang [1], Siyuan Shen [1], Jinmiao Song [2,3], and Lei Deng [1,2,3,*]

*To whom correspondence should be addressed.
†These authors contributed equally to this work.

[1]School of Computer Science and Engineering, Central South University, Changsha, 410083, China
[2]Xinjiang Key Laboratory of Intelligent Computing and Smart Applications, Xinjiang University, Urumqi, 830046, China
[3]School of Software, Xinjiang University, Urumqi, 830046, China

Contents

1 Cross-attention for interaction modeling **3**

2 Evidence modeling and gating strategy **5**

3 Data curation and preprocessing **7**

4 Baseline methods and evaluation protocol **8**

5 Supplementary Experiment on Pocket-Augmented PROTAC-STAN **10**

6 Interpretability Analysis and Additional Visualizations **11**

7 Strict cold-start evaluation on Protac3656 **14**

8 Parameter sensitivity analysis **16**

9 Supplementary Analysis of KLHDC2-Based BET PROTACs **17**

10 Calibration Analysis **19**

11 Model Parameter Count **20**

12 Supplementary Experiment on Pocket Mask Source **21**

1 Cross-attention for interaction modeling

This section provides implementation details for the cross-attention interaction module summarized in the main text. Pocket-PROTACs models two protein–molecule interaction branches in parallel, namely POI–PROTAC and E3–PROTAC, using multi-head scaled dot-product attention where protein residues attend to PROTAC atoms. Let $H_P^d \in \mathbb{R}^{B\times L_P\times d}$ and $H_E^d \in \mathbb{R}^{B\times L_E\times d}$ denote the projected residue embeddings for POI and E3 ligase, and let $A \in \mathbb{R}^{B\times N\times d}$ denote the padded atom embeddings of the PROTAC (after conformer fusion), where B is the batch size, L_P/L_E are padded sequence lengths, and N is the padded atom length.

Multi-head cross-attention. We apply two cross-attention blocks:

$$C_P = \mathrm{Attn}(H_P^d, A, A), \qquad C_E = \mathrm{Attn}(H_E^d, A, A), \tag{1}$$

where $C_P \in \mathbb{R}^{B\times L_P\times d}$ and $C_E \in \mathbb{R}^{B\times L_E\times d}$. The module returns both the attended residue features and attention weights:

$$W_P \in \mathbb{R}^{B\times L_P\times N}, \qquad W_E \in \mathbb{R}^{B\times L_E\times N}, \tag{2}$$

where the weights are averaged over heads. Following attention, we apply layer normalization to stabilize training:

$$C_P \leftarrow \mathrm{LN}(C_P), \qquad C_E \leftarrow \mathrm{LN}(C_E). \tag{3}$$

Learnable attention pooling for interaction embeddings. As described in the main text, residue-level interaction features are summarized into fixed-size embeddings $b_P, b_E \in \mathbb{R}^{B\times d}$ via learnable attention pooling. Concretely, for a residue tensor $C \in \mathbb{R}^{B\times L\times d}$ and a validity mask $M \in \{0,1\}^{B\times L}$ (1 indicates a real residue, 0 indicates padding), we compute a scalar score per residue using a small scoring network $s(\cdot)$:

$$\tilde{\alpha}_i = s(C_i), \quad i = 1, \dots, L, \tag{4}$$

then apply mask-aware softmax normalization across residues:

$$\alpha_i = \frac{\exp(\tilde{\alpha}_i)\cdot M_i}{\sum_{k=1}^{L}\exp(\tilde{\alpha}_k)\cdot M_k}, \tag{5}$$

and finally pool:

$$b = \sum_{i=1}^{L} \alpha_i \, C_i. \tag{6}$$

We apply the above pooling to C_P and C_E to obtain b_P and b_E, respectively. In our implementation, $s(\cdot)$ is a two-layer MLP with GELU nonlinearity producing a single logit per residue.

Residue importance from attention weights. To support pocket-focused aggregation and evidence alignment in later stages, we derive residue-level importance vectors $a_P \in \mathbb{R}^{B \times L_P}$ and $a_E \in \mathbb{R}^{B \times L_E}$ from the cross-attention weights. Intuitively, a measures how strongly each residue attends to any molecular region. Specifically, for each residue we take the maximum attention over atoms and then normalize across valid residues:

$$\tilde{a}_i = \max_{1 \le j \le N} W_{ij}, \qquad a_i = \frac{\tilde{a}_i \cdot M_i}{\sum_{k=1}^{L} \tilde{a}_k \cdot M_k}, \tag{7}$$

where M is the residue validity mask. This produces nonnegative weights that sum to 1 over real residues, consistent with the normalization used later for pocket coverage.

2 Evidence modeling and gating strategy

This section details the evidence modeling and cooperative gating strategy summarized in the main text. The goal is twofold: (i) quantify confidence of interaction embeddings derived from cross-attention, and (ii) amplify cooperative interaction signals when both POI–PROTAC and E3–PROTAC interactions are simultaneously supported.

Pocket coverage from residue importance. Given residue importance vectors $a_P \in \mathbb{R}^{B\times L_P}$ and $a_E \in \mathbb{R}^{B\times L_E}$ (Section 1), we compute pocket coverage scores using pocket masks $m_P \in \{0,1\}^{B\times L_P}$ and $m_E \in \{0,1\}^{B\times L_E}$ (1 indicates a residue belongs to the pocket/interface set; 0 otherwise). Pocket coverage is defined as:

$$r_P = \sum_{i=1}^{L_P} a_{P,i}\, m_{P,i}, \qquad r_E = \sum_{i=1}^{L_E} a_{E,i}\, m_{E,i}, \tag{8}$$

which yields $r_P, r_E \in [0,1]$.

Confidence-aware evidence vectors. Let $b_P, b_E \in \mathbb{R}^{B\times d}$ be the pooled interaction embeddings produced by attention pooling. We map each b to an evidence vector $e \in (0,1)^{B\times d}$ using a learnable linear transform followed by element-wise sigmoid gating:

$$e_P = \sigma(\phi_P(b_P))\,, \qquad e_E = \sigma(\phi_E(b_E))\,, \tag{9}$$

where $\phi_P, \phi_E : \mathbb{R}^d \to \mathbb{R}^d$ are learnable affine maps and $\sigma(\cdot)$ is the sigmoid function. We also summarize evidence strength by the mean evidence score:

$$s_P = \frac{1}{d}\sum_{k=1}^{d} e_{P,k}, \qquad s_E = \frac{1}{d}\sum_{k=1}^{d} e_{E,k}. \tag{10}$$

Pocket alignment loss. To encourage mechanistically plausible attribution, we align the evidence strength with pocket-focused attention via:

$$\mathcal{L}_{\text{align}} = \mathbb{E}\left[(s_P - r_P)^2 + (s_E - r_E)^2\right], \tag{11}$$

which penalizes confident evidence not supported by pocket-localized residue importance.

Evidence regularization. To avoid overly saturated evidence gates and improve numerical stability, we add a smoothness regularizer:

$$\mathcal{L}_{\text{evid}} = \mathbb{E}\left[\frac{1}{d}\sum_{k=1}^{d} e_{P,k}(1-e_{P,k}) + \frac{1}{d}\sum_{k=1}^{d} e_{E,k}(1-e_{E,k})\right]. \tag{12}$$

Cooperative gating and fusion. We model cooperative interaction by gating the combined interaction embedding using the agreement of e_P and e_E. First, we combine the two interaction embeddings:

$$u_0 = b_P + b_E \in \mathbb{R}^{B\times d}. \tag{13}$$

We then compute an evidence agreement score as the mean element-wise product:

$$g = \frac{1}{d}\sum_{k=1}^{d} e_{P,k}\, e_{E,k} \in \mathbb{R}^{B}. \tag{14}$$

A nonnegative sample-specific scaling factor β is predicted from the global molecular representation z_M:

$$\beta = \text{softplus}(w^\top z_M + b) \in \mathbb{R}^{B}_{\geq 0}. \tag{15}$$

The final cooperative gate is:

$$\text{gate} = 1 + \beta \cdot g, \tag{16}$$

and the gated cooperative interaction embedding is:

$$u = u_0 \odot \text{gate}, \tag{17}$$

where $\odot$ denotes channel-wise scaling (broadcasting `gate` over the d dimensions).

Final prediction head. Finally, we fuse the gated interaction u with global protein and molecular context. Let $z_P^d, z_E^d \in \mathbb{R}^{B\times d}$ be projected global protein embeddings and $z_M \in \mathbb{R}^{B\times d_M}$ be the global molecular embedding. We compute:

$$t_0 = \psi([z_P^d \| z_E^d \| z_M]), \qquad \hat{y} = \sigma\left(h([u\|t_0])\right), \tag{18}$$

where $\psi(\cdot)$ and $h(\cdot)$ are MLPs, and $\hat{y} \in (0,1)$ is the predicted degradation probability. The overall training objective is:

$$\mathcal{L} = \mathcal{L}_{\text{cls}} + \lambda_{\text{align}}\mathcal{L}_{\text{align}} + \lambda_{\text{evid}}\mathcal{L}_{\text{evid}}, \tag{19}$$

where $\mathcal{L}_{\text{cls}}$ is binary cross-entropy with logits.

3 Data curation and preprocessing

All datasets were curated using explicit rule-based criteria to ensure that each retained sample constituted a valid PROTAC triplet (P, E, M), where P denotes the target protein, E the recruited E3 ligase, and M the PROTAC molecule. Entries were discarded if the target protein or E3 ligase lacked a valid UniProt accession, if no experimental degradation-related measurement was available, or if the PROTAC molecular structure could not be unambiguously parsed from the reported SMILES representation. Duplicate records corresponding to identical PROTAC–target–E3 combinations were merged to avoid redundancy.

Binary degradation labels were assigned using a unified hierarchical strategy to accommodate heterogeneous experimental readouts. When available, DC50 values were used as the primary criterion, with samples classified as active if DC50 was below 1000 nM. If DC50 was unavailable, degradation percentage measurements were used, with values exceeding 70% considered indicative of degradation. In the absence of both DC50 and degradation percentage, IC50 values were used with the same 1000 nM threshold. Samples lacking all three types of measurements were excluded from further analysis. For each retained triplet, amino-acid sequences of the target protein and E3 ligase were retrieved from UniProt [1] and encoded using a pre-trained protein language model. To enable pocket-aware modeling, binding-pocket information was incorporated using experimentally annotated pockets when available, or predicted pocket regions obtained from P2Rank [2] otherwise. PROTAC molecular structures were obtained from curated SMILES strings in PROTAC-DB, and three-dimensional conformer ensembles were generated using RDKit [3] to support geometry-aware molecular representation learning.

To rigorously evaluate model generalization to unseen PROTAC molecules, a molecule-disjoint data splitting strategy was adopted following prior PROTAC degradation prediction studies [4, 5]. For each dataset, PROTAC molecules were partitioned into training and test sets at an 8:2 ratio, ensuring that no PROTAC molecule present in the test set was observed during training. All samples associated with the same PROTAC molecule were assigned to the same split, thereby preventing information leakage. To mitigate the influence of random partitioning and improve statistical robustness, this splitting procedure was repeated multiple times using different random seeds.

4 Baseline methods and evaluation protocol

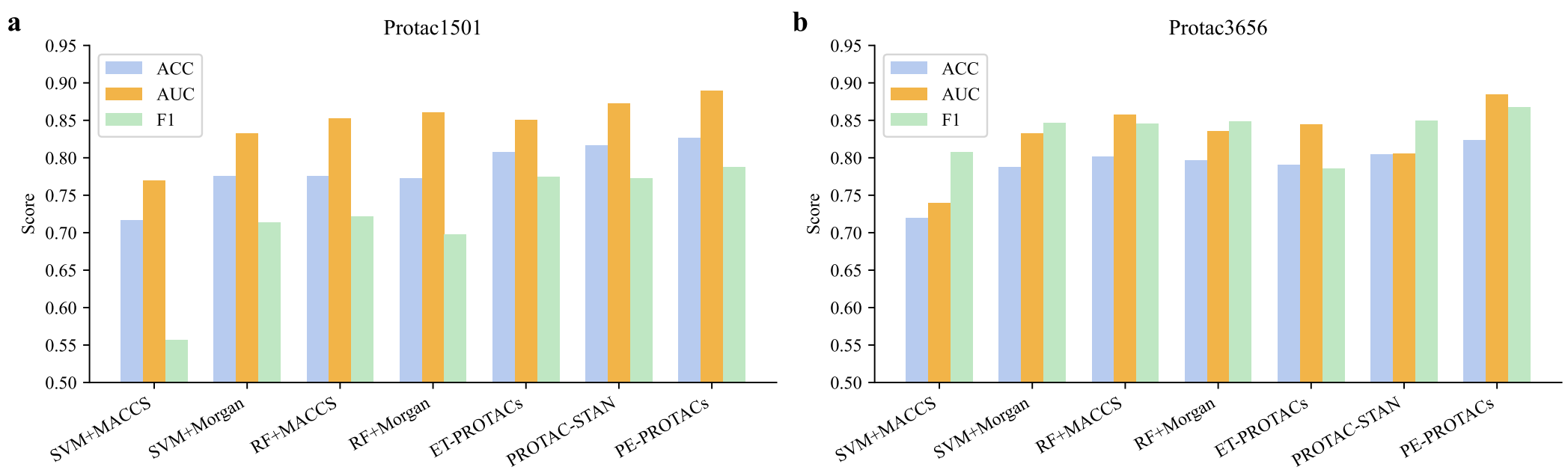

Figure S1: Performance comparison of different methods on the Protac1501 and Protac3656 datasets. Panels (a) and (b) correspond to Protac1501 and Protac3656, respectively. For each method, ACC, AUC, and F1-score are reported.

We compared Pocket-PROTACs with representative deep learning and classical machine learning baselines on both datasets. All baseline methods were evaluated under the same experimental settings, and performance was reported using AUC, accuracy (ACC), F1-score, precision (Pre), and recall (Rec). Fig. S1 complements Table 1 in the main text by visualizing the performance of selected baselines on Protac1501 and Protac3656 under the same 8:2 split.

- **DNN + fingerprints**: A multilayer perceptron classifier operating on fixed-length molecular fingerprint representations of PROTACs, including Morgan or MACCS fingerprints.
- **RF + fingerprints** [6]: A Random Forest classifier using handcrafted chemical descriptors, where PROTAC molecules are encoded by Morgan [7] or MACCS [8] fingerprints, serving as a strong traditional machine learning baseline.
- **SVM + fingerprints** [9]: A Support Vector Machine classifier trained on the same Morgan or MACCS fingerprint representations, providing an additional classical baseline.
- **Transformer + fingerprints** [10]: A Transformer-based model that encodes PROTAC molecules from tokenized SMILES strings using self-attention, whose learned sequence representations are further fused with molecular fingerprint features via late fusion for classification.

- **PROTAC-STAN** [4]: A structure-informed deep learning framework that models PROTAC-induced degradation via ternary attention, explicitly capturing interactions among the POI, E3 ligase, and PROTAC molecule while providing residue- and atom-level interpretability.
- **ET-PROTACs** [5]: A cross-modal learning approach that integrates 3D molecular representations of PROTACs with protein features using a ternary attention mechanism to model cooperative interactions within the ternary complex.

5 Supplementary Experiment on Pocket-Augmented PROTAC-STAN

To further investigate the contribution of pocket information to PROTAC-induced degradation prediction, we constructed a pocket-augmented variant of PROTAC-STAN, denoted as PROTAC-STAN+Pocket. In this variant, pocket information is incorporated into PROTAC-STAN by concatenating pocket-level representations to the protein embeddings prior to the ternary attention module, while keeping all other components of PROTAC-STAN unchanged. Specifically, for each protein, we extract pocket residue embeddings using the same pre-trained protein language model employed in Pocket-PROTACs, producing a 1280-dimensional pocket embedding, along with a 30-dimensional physicochemical feature vector summarizing pocket properties. These are concatenated with the original protein representation and projected back to the model hidden dimension via a linear layer. All other training settings, including dataset splits, random seeds, and hyperparameters, are kept identical to ensure a fair comparison.

Table S1: Performance comparison of PROTAC-STAN, PROTAC-STAN+Pocket, and Pocket-PROTACs on the Protac3656 dataset.

Method	AUC	ACC	F1	Pre	Rec
PROTAC-STAN	0.8058	0.8046	0.8496	0.8360	0.8581
PROTAC-STAN+Pocket	0.8312	0.8134	0.8561	0.8412	0.8693
Pocket-PROTACs	**0.8844**	**0.8237**	**0.8675**	**0.8500**	**0.8864**

As shown in Table S1, PROTAC-STAN+Pocket achieves moderate improvements over the base PROTAC-STAN across most metrics, suggesting that pocket annotations can provide useful structural signals even when incorporated through simple feature concatenation. Pocket-PROTACs further improves upon PROTAC-STAN+Pocket, which may indicate that the pocket-aware attention alignment mechanism allows pocket information to be more effectively utilized during interaction modeling. These results collectively highlight the value of pocket information for PROTAC degradation prediction, and suggest that architectural choices for incorporating such information may also play a role in determining its impact.

6 Interpretability Analysis and Additional Visualizations

In the main text, atom-level importance is visualized separately for the E3–PROTAC and POI–PROTAC interaction branches. The E3 attention map concentrates around the glutarimide-like E3-recruiting moiety (atoms 27, 28, 31), while the POI attention map shows a broader distribution centered on the POI-binding warhead (atoms 1, 52) with additional contributions from linker-adjacent atoms. A partial overlap between the two maps is observed around certain linker and junction regions, which has a structural basis: linker atoms occupy a central position in the PROTAC scaffold and may simultaneously contribute to the spatial arrangement of both binding ends within the ternary complex. In both maps, linker atoms also receive moderate attention, suggesting that the model captures the cooperative role of the linker rather than treating it as a passive connector.

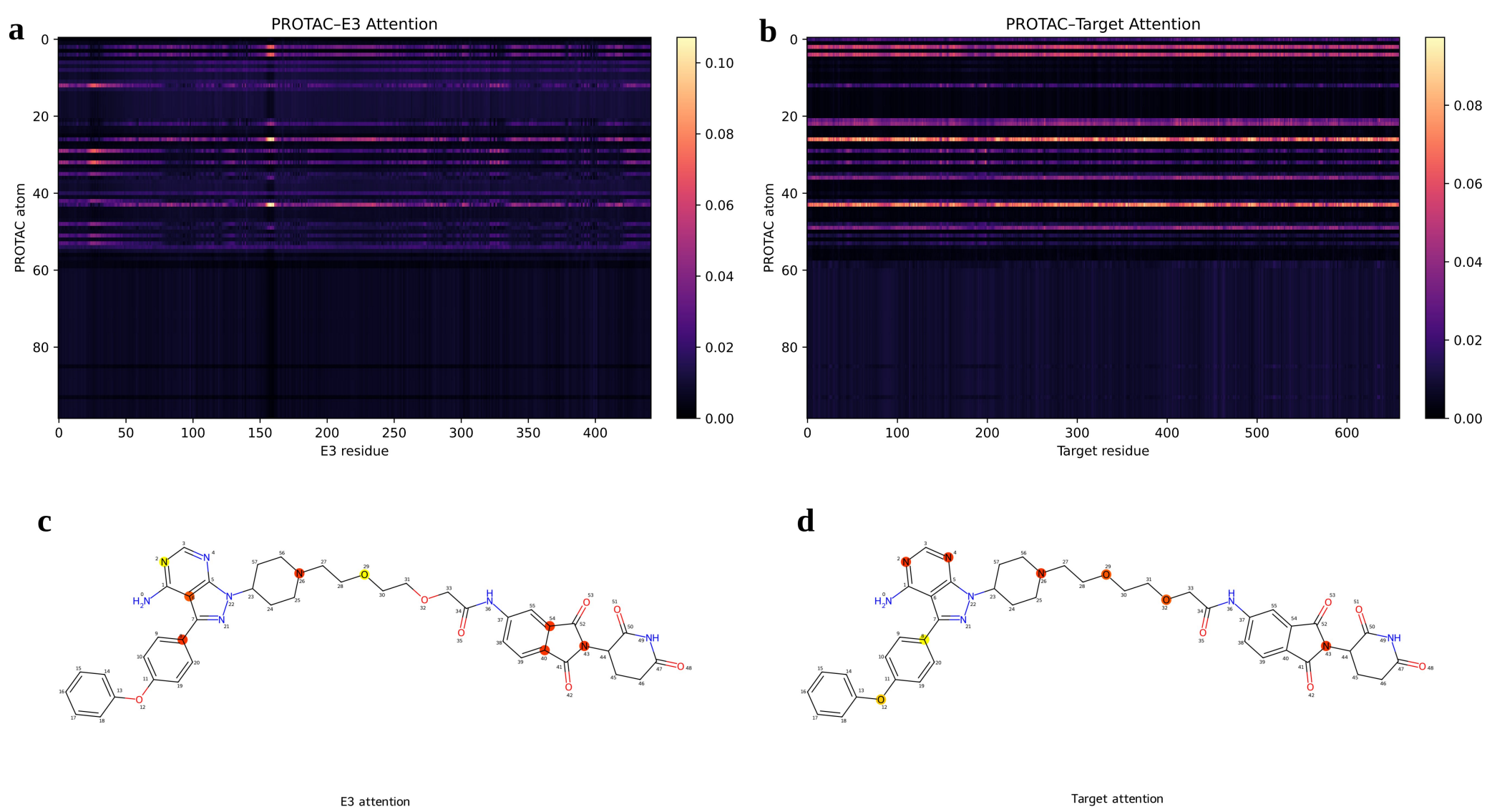

Figure S2: Atom-level attention visualization for case idx140 (PROTAC CID283, POI: Q06187, E3: Q96SW2). (a) PROTAC–E3 attention heatmap. (b) PROTAC–Target attention heatmap. (c) E3 attention map on the 2D PROTAC structure. (d) POI attention map on the 2D PROTAC structure.

To provide a more systematic assessment of the learned atom-level attention patterns, we present three additional visualization examples from the test set, covering PROTAC molecules targeting different POI proteins (UniProt: Q06187, Q00534,

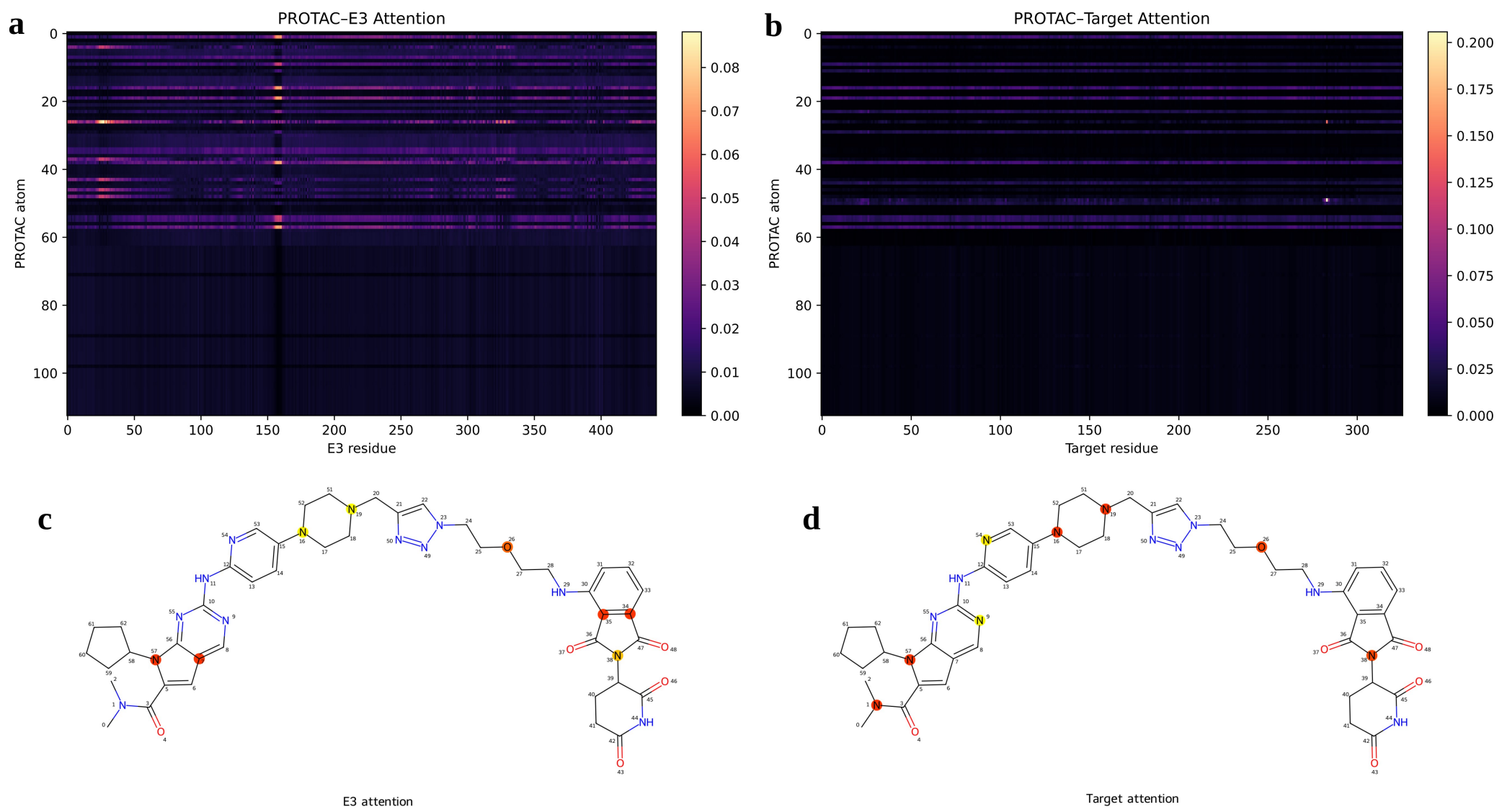

Figure S3: Atom-level attention visualization for case idx250 (PROTAC CID424, POI: Q00534, E3: Q96SW2). (a) PROTAC–E3 attention heatmap. (b) PROTAC–Target attention heatmap. (c) E3 attention map on the 2D PROTAC structure. (d) POI attention map on the 2D PROTAC structure.

Q05397) in combination with the same E3 ligase (Q96SW2). For each case, E3-PROTAC and POI-PROTAC atom importance maps are shown separately, with the top-12 atoms ranked by attention highlighted using the same color scheme as in the main text (red to orange to yellow, indicating decreasing importance).

As shown in Figs. S2–S4, several consistent trends emerge across all three cases. First, the E3 attention maps consistently assign high importance to atoms localized around the E3-recruiting moiety, particularly the glutarimide-like ring system that mediates CRBN engagement. This recurring pattern across structurally diverse PROTACs supports the interpretation that the model has learned a stable and chemically meaningful representation of E3 ligand recognition, consistent with the well-characterized binding mode of CRBN recruiters. Second, the POI attention maps display a more compound-specific distribution, with high-importance atoms concentrated around the POI-binding warhead while also involving selected linker-adjacent atoms. The warhead-centric attention is consistent with the expectation that the POI-binding end primarily determines target selectivity, while the greater variability across cases reflects the structural diversity of warheads targeting different POI proteins.

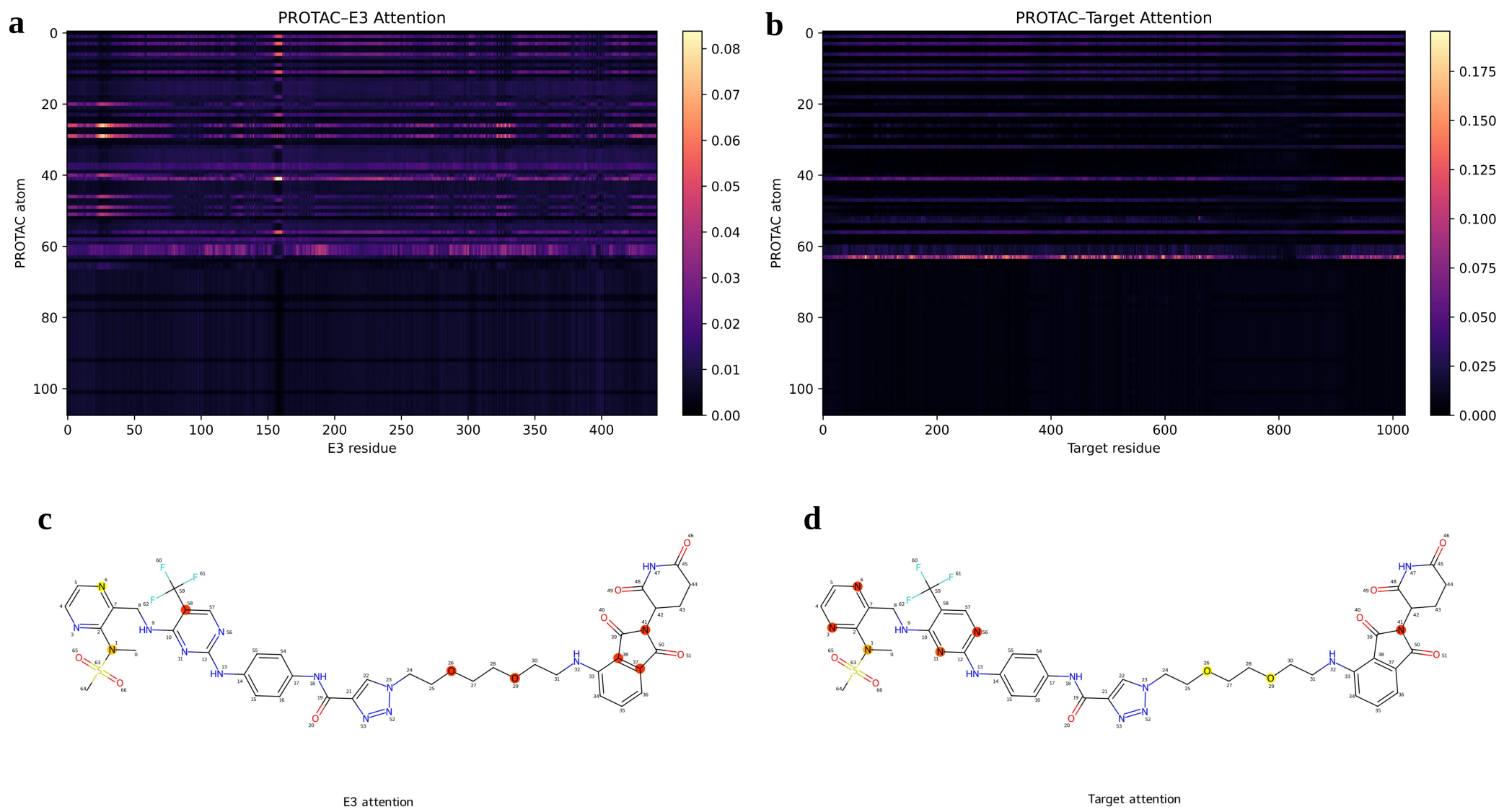

Figure S4: Atom-level attention visualization for case idx820 (PROTAC CID1813, POI: Q05397, E3: Q96SW2). (a) PROTAC–E3 attention heatmap. (b) PROTAC–Target attention heatmap. (c) E3 attention map on the 2D PROTAC structure. (d) POI attention map on the 2D PROTAC structure.

A partial overlap between the E3 and POI attention maps is observed in some cases, particularly around linker junction regions. This pattern has a structural basis: linker atoms occupy a central position in the PROTAC scaffold and play a role in determining the geometry and flexibility of the ternary complex, potentially contributing to the interaction signals captured by both branches. The degree of overlap varies across cases, suggesting that the model adapts its attention distribution to the specific structural context of each PROTAC rather than uniformly conflating the two branches. Taken together, these additional examples confirm that the atom-level attention patterns learned by Pocket-PROTACs are chemically interpretable, consistent across diverse PROTAC scaffolds, and reflective of the bifunctional binding mechanism underlying PROTAC-induced degradation.

7 Strict cold-start evaluation on Protac3656

To further assess model generalization under realistic deployment scenarios, we conducted an additional strict cold-start evaluation on the Protac3656 dataset. In this setting, we first enforce a POI-disjoint split, ensuring that POI proteins (identified by UniProt IDs) in the test set do not appear in the training set. We then apply a second-stage filtering step to the test set by removing any PROTAC compounds whose SMILES strings have appeared in the training data. Consequently, the final test set satisfies a stringent constraint in which both POIs and PROTAC compounds are completely unseen during training, substantially reducing potential information leakage and resulting in a more challenging evaluation protocol. Under this strict cold-start setting, we compare Pocket-PROTACs with two representative baselines, ET-PROTACs and PROTAC-STAN, which demonstrate strong performance in the standard evaluation. The cold-start results on Protac3656 are summarized in Tbale. S2 and Fig. S5.

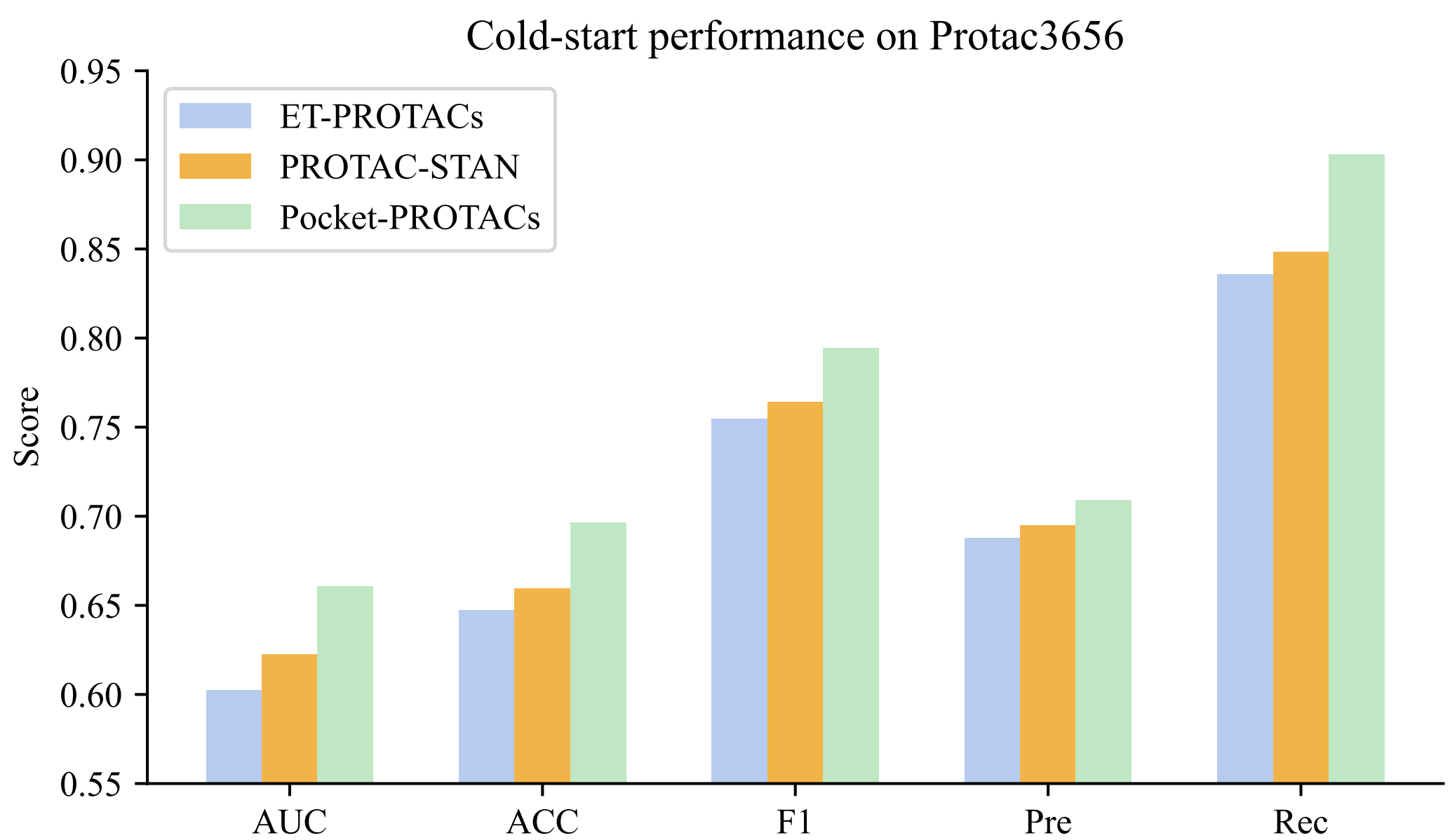

Figure S5: Cold-start performance comparison on the Protac3656 dataset.

As shown in Tbale. S2, Pocket-PROTACs consistently outperforms both baselines across all evaluation metrics, including AUC, ACC, F1-score, Precision, and Recall. In particular, Pocket-PROTACs achieves a higher AUC (0.6605) compared with ET-

Table S2: Performance comparison under the cold-start setting on the Protac3656 dataset.

Method	AUC	ACC	F1	Pre	Rec
ET-PROTACs	0.6024	0.6471	0.7548	0.6880	0.8358
PROTAC-STAN	0.6223	0.6594	0.7640	0.6948	0.8484
Pocket-PROTACs	**0.6605**	**0.6963**	**0.7944**	**0.7091**	**0.9032**

PROTACs (0.6024) and PROTAC-STAN (0.6223), indicating improved discrimination capability under severe distribution shifts. Moreover, Pocket-PROTACs achieves the highest recall (0.9032) while maintaining competitive precision (0.7091), suggesting enhanced sensitivity to true degraders without sacrificing specificity. Overall, these results demonstrate that Pocket-PROTACs exhibits stronger robustness and generalization ability under a strict entity-level cold-start setting, highlighting the advantage of cross-modal interaction modeling when both POIs and PROTAC compounds are out-of-distribution.

8 Parameter sensitivity analysis

We further analyze the sensitivity of Pocket-PROTACs to several key hyperparameters, including the learning rate, the depth of the EGNN module, and the number of attention heads (Fig. S6). Overall, the model exhibits stable performance trends across all three metrics (AUC, ACC, and F1-score) within reasonable parameter ranges, indicating that the proposed architecture does not rely on finely tuned hyperparameters to achieve strong predictive performance.

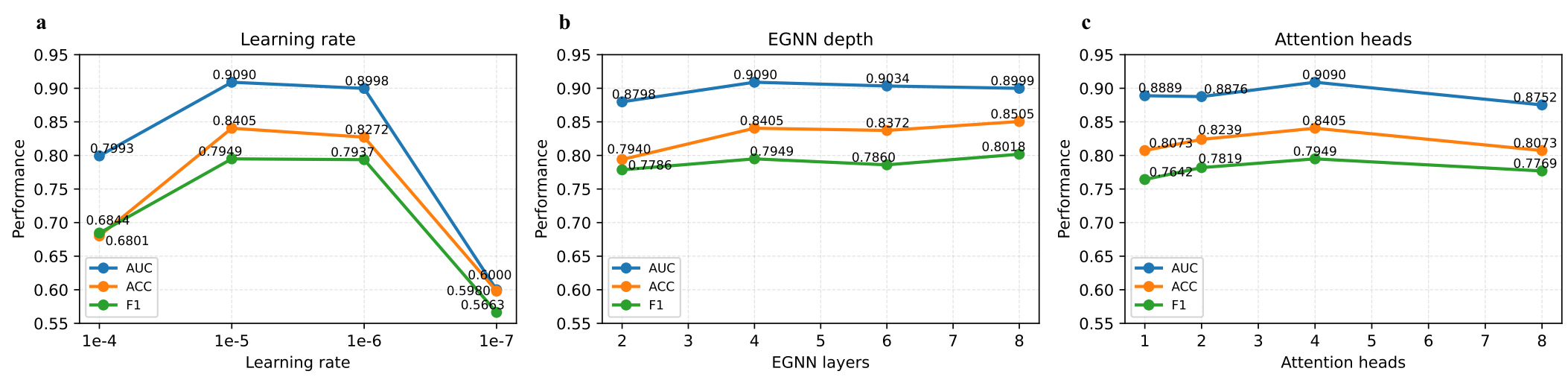

Figure S6: Parameter sensitivity analysis of Pocket-PROTACs under the unseen-molecule setting. The effects of learning rate, EGNN depth, and the number of attention heads are evaluated in terms of AUC, ACC, and F1-score.

As shown in Fig. S6(a), the learning rate has a pronounced impact on model behavior. The best performance is achieved at 1×10^{-5}, while both larger and smaller values lead to performance degradation. In particular, an excessively small learning rate results in a sharp drop across all metrics, suggesting insufficient optimization of the multi-component architecture. Fig. S6(b) shows that increasing the EGNN depth from 2 to 4 improves performance, whereas deeper configurations provide limited additional gains, indicating diminishing returns from overly deep geometric message passing. A similar trend is observed for the number of attention heads in Fig. S6(c), where a moderate setting (4 heads) yields the best overall results, while fewer or more heads lead to reduced performance. These observations support the default hyperparameter configuration adopted in this work and demonstrate that Pocket-PROTACs operates in a stable and well-balanced regime.

9 Supplementary Analysis of KLHDC2-Based BET PROTACs

To assess model performance on experimentally validated systems, we conducted a case study based on a series of KLHDC2-engaging PROTACs reported by Scott et al. [11]. This system provides a controlled setting for paralog-selective targeted protein degradation, characterizing the responses of three highly homologous BET family members (BRD2, BRD3, and BRD4) under recruitment of a single E3 ligase. The experimental system comprises four structurally related PROTACs, SJ46420, SJ46421, SJ48087, and SJ48088. All compounds share a common BET-binding warhead and a KLHDC2-recruiting moiety, while differing in linker composition and terminal chemical modifications, leading to distinct degradation behaviors across BRD paralogs.

Quantitative evaluation was based on experimentally measured degradation data from the KLHDC2 knockout and rescue system at a fixed concentration of 1 μM. Under this condition, SJ46420, SJ48087, and SJ48088 were evaluated against all three BET targets. SJ46421 was not profiled in the knockout–rescue system; however, under the U2OS 5 μM condition, it consistently showed near-baseline protein levels across all BRD paralogs and was therefore included as an inactive control for classification-level evaluation. Degradation labels were derived from normalized protein abundance measured by Western blot, with targets exhibiting less than 50% remaining protein assigned as degraded.

Table S3: Experimental normalized protein levels and model predictions for the KLHDC2-engaging BRD case study. Columns BRD2/3/4 (%) show normalized protein abundance, while True (2/3/4) and Pred (2/3/4) indicate experimental binary labels and model predictions for BRD2, BRD3, and BRD4, respectively.

PROTAC	Condition	BRD2/3/4 (%)	True (2/3/4)	Pred (2/3/4)
SJ46420	KO/Rescue, 1 μM	88/45/90	0/1/0	0/1/0
SJ48087	KO/Rescue, 1 μM	90/89/101	0/0/0	0/0/0
SJ48088	KO/Rescue, 1 μM	83/46 79	0/1/0	1/1/0
SJ46421	U2OS, 5 μM	127/114/111	0/0/0	0/0/0

From this system, we constructed a total of 12 PROTAC–target cases, correspond-

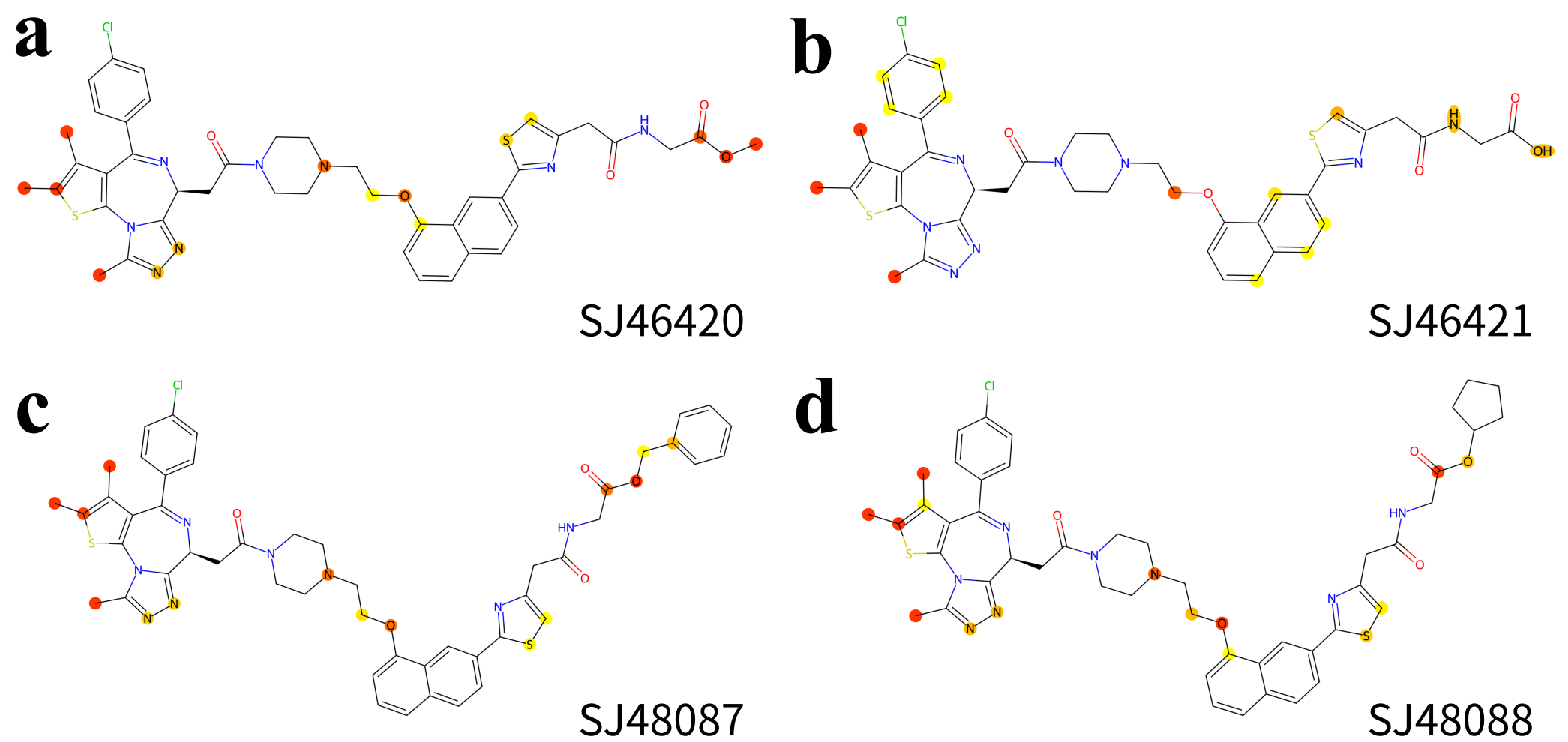

Figure S7: 2D structures of four PROTAC variants used in the case study: (a) SJ46420, (b) SJ46421, (c) SJ48087, and (d) SJ48088.

ing to all combinations between four PROTACs and three BET paralogs. Table S3 summarizes the experimental readouts, derived binary labels, model predictions. To further interpret the model's predictions, we visualized the fused cross-attention on the four PROTACs (Fig. S7). The attention scores are obtained by integrating cross-attention from both interaction branches (PROTAC-POI and PROTAC-E3 ligand) and mapped onto PROTAC atoms. The coloring scheme follows the same convention as in Fig. 3(e) in the main text, while the visualization highlights the top-15 atoms ranked by fused attention. Across all four PROTACs, a consistent attention pattern is observed: high attention is concentrated at both molecular termini, the POI-binding warhead and the E3-ligand moiety, as well as on several key atoms along the linker, particularly near junctions such as heteroatoms or amide and carbonyl connections. This distribution indicates that the model integrates contributions from both binding ends together with linker-associated atoms, rather than relying on a single localized fragment. Although the four PROTACs share the same warhead and overall linker scaffold, the most pronounced compound-specific differences in attention are localized to the E3-ligand end, which represents the primary structural variation within this series. While the conserved backbone exhibits broadly similar attention patterns, the emphasized atoms within the E3-ligand moiety vary across compounds, suggesting that these regions contribute to the model's discriminative signals.

10 Calibration Analysis

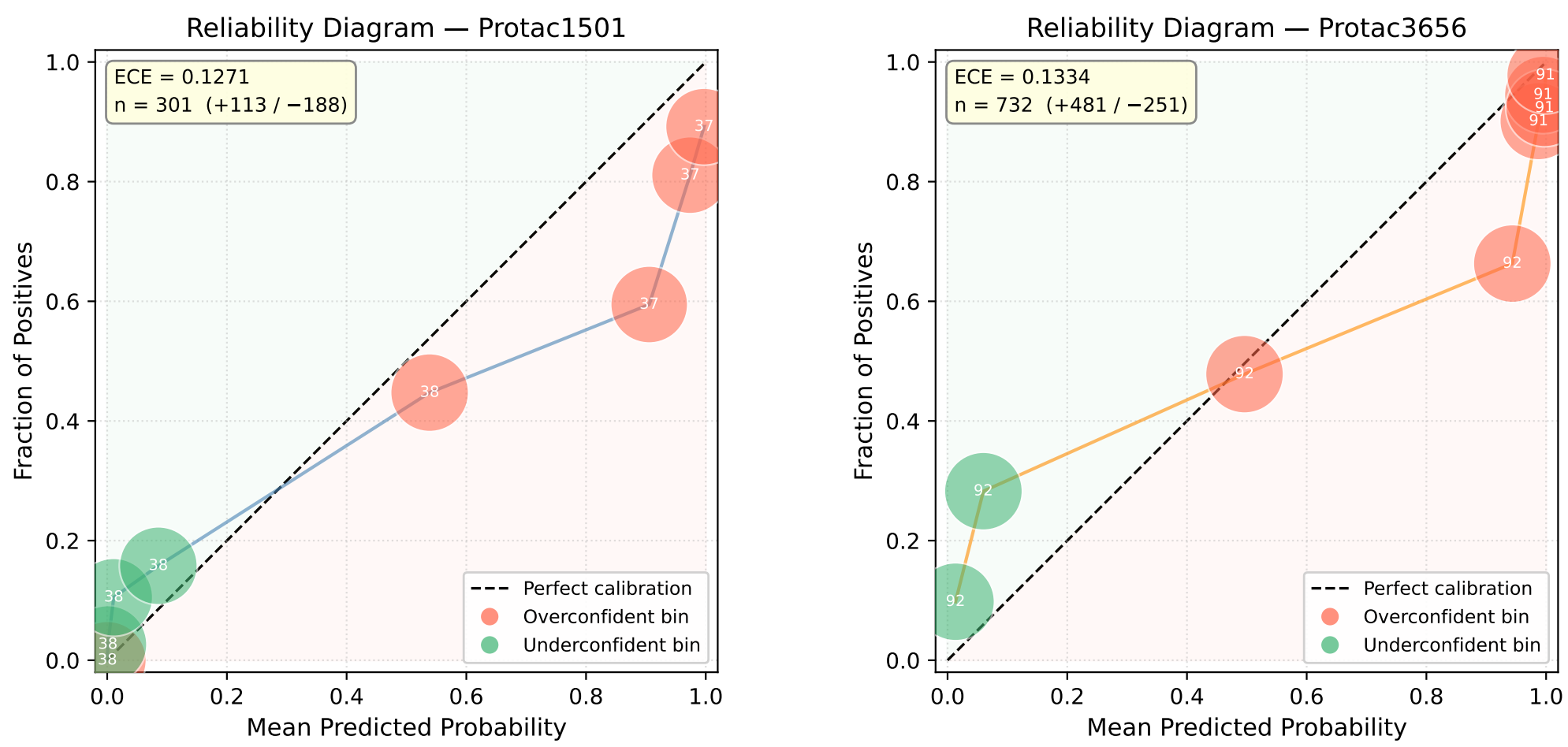

Figure S8: Reliability diagrams for Pocket-PROTACs on Protac1501 (left) and Protac3656 (right). Each point represents a probability bin. Red and green points indicate overconfident and underconfident bins, respectively.

To assess the reliability of predicted probabilities, we performed a calibration analysis on both datasets using reliability diagrams and Expected Calibration Error (ECE). The ECE measures the average discrepancy between predicted probabilities and observed positive rates across equal-frequency bins. As shown in Fig. S8, the ECE values are 0.1271 on Protac1501 and 0.1334 on Protac3656. The reliability diagrams reveal a polarized pattern: samples assigned low predicted probabilities tend to be underconfident (actual positive rate lower than predicted), while those assigned high predicted probabilities tend to be overconfident. This behavior is characteristic of binary classifiers with strong discriminative power, where the model assigns confident predictions near 0 or 1. While this suggests that the raw predicted probabilities should be interpreted with caution as absolute confidence estimates, the discriminative performance as measured by AUC remains unaffected. Post-hoc calibration techniques such as temperature scaling may be explored in future work to improve probability reliability.

11 Model Parameter Count

Table S4 reports the number of trainable parameters in each module of Pocket-PROTACs, with a total of 3,891,335 parameters.

Table S4: Trainable parameter count by module in Pocket-PROTACs.

Module	Parameters	% of Total
EGNN molecular encoder (4 layers)	1,317,892	33.9%
Protein projection	656,384	16.9%
Global context MLP	787,456	20.2%
Cross-attention modules ($\times$2) + LayerNorm	527,360	13.6%
Props MLP + z_M projection	214,912	5.5%
Prediction head	197,121	5.1%
Evidence heads ($\times$2)	131,584	3.4%
Attention pooling	33,025	0.8%
Atom embedding + projection	24,832	0.6%
Conformer attention + Beta gate	769	$<$0.1%
**Total**	**3,891,335**	**100%**

12 Supplementary Experiment on Pocket Mask Source

To further motivate the use of externally annotated pocket masks as structural priors, we investigate whether a simpler alternative can achieve comparable performance. Specifically, we construct a variant termed **DataDrivenMask**, in which the externally provided pocket masks are replaced by soft weights inferred from the cross-attended residue representations via a learned scoring function, with no access to any structural annotation. Unlike the full Pocket-PROTACs, which relies on experimentally annotated or P2Rank-predicted pocket residues to guide evidence alignment, DataDrivenMask attempts to discover relevant residues purely from data. All other components remain identical to the full model.

Table S5: Comparison of pocket mask sources on the Protac3656 dataset.

Variant	AUC	ACC	F1	Pre	Rec
NoPocket	0.8762	0.8040	0.7631	0.7143	0.8190
DataDrivenMask	0.8829	0.8115	0.7693	0.7198	**0.8261**
Pocket-PROTACs	**0.9090**	**0.8405**	**0.7949**	**0.7686**	0.8230

As shown in Table S5, DataDrivenMask consistently underperforms Pocket-PROTACs across all primary metrics, with the largest gaps observed in AUC and precision, indicating that the benefit of pocket-aware modeling stems from the structural prior rather than from adding a learnable component. Notably, DataDrivenMask achieves a marginally higher recall (0.8261), yet this does not improve overall discrimination. These results collectively highlight that the quality and origin of pocket information matter, and justify incorporating externally derived pocket masks as structural priors in Pocket-PROTACs.

References

[1] UniProt Consortium. Uniprot: a worldwide hub of protein knowledge. *Nucleic acids research*, 47(D1):D506–D515, 2019.

[2] Radoslav Krivák and David Hoksza. P2rank: machine learning based tool for rapid and accurate prediction of ligand binding sites from protein structure. *Journal of cheminformatics*, 10(1):39, 2018.

[3] Greg Landrum. Rdkit: Open-source cheminformatics. *Available at https://www.rdkit.org*, 2013.

[4] Zhenglu Chen, Chunbin Gu, Shuoyan Tan, Xiaorui Wang, Yuquan Li, Mutian He, Ruiqiang Lu, Shijia Sun, Chang-Yu Hsieh, Xiaojun Yao, et al. Interpretable protac degradation prediction with structure-informed deep ternary attention framework. *Advanced Science*, 12(47):e08138, 2025.

[5] Lijun Cai, Guanyu Yue, Yifan Chen, Li Wang, Xiaojun Yao, Quan Zou, Xiangzheng Fu, and Dongsheng Cao. Et-protacs: modeling ternary complex interactions using cross-modal learning and ternary attention for accurate protac-induced degradation prediction. *Briefings in Bioinformatics*, 26(1):bbae654, 2025.

[6] Leo Breiman. Random forests. *Machine learning*, 45(1):5–32, 2001.

[7] Shifa Zhong and Xiaohong Guan. Count-based morgan fingerprint: A more efficient and interpretable molecular representation in developing machine learning-based predictive regression models for water contaminants' activities and properties. *Environmental science & technology*, 57(46):18193–18202, 2023.

[8] DJ Polton. Installation and operational experiences with maccs (molecular access system). *Online Review*, 6(3):235–242, 1982.

[9] Marti A. Hearst, Susan T Dumais, Edgar Osuna, John Platt, and Bernhard Scholkopf. Support vector machines. *IEEE Intelligent Systems and their applications*, 13(4):18–28, 1998.

[10] Ashish Vaswani, Noam Shazeer, Niki Parmar, Jakob Uszkoreit, Llion Jones, Aidan N Gomez, Łukasz Kaiser, and Illia Polosukhin. Attention is all you need. *Advances in neural information processing systems*, 30, 2017.

[11] Daniel C Scott, Suresh Dharuman, Elizabeth Griffith, Sergio C Chai, Jarrid Ronnebaum, Moeko T King, Rajendra Tangallapally, Chan Lee, Clifford T Gee, Lei Yang, et al. Principles of paralog-specific targeted protein degradation engaging the c-degron e3 klhdc2. *Nature Communications*, 15(1):8829, 2024.
